# CD36, a signaling receptor and fatty acid transporter that regulates immune cell metabolism and fate

**DOI:** 10.1084/jem.20211314

**Published:** 2022-04-19

**Authors:** Yiliang Chen, Jue Zhang, Weiguo Cui, Roy L. Silverstein

**Affiliations:** 1 Department of Medicine, Medical College of Wisconsin, Milwaukee, WI; 2 Versiti, Blood Research Institute, Milwaukee, WI; 3 Department of Microbiology and Immunology, Medical College of Wisconsin, Milwaukee, WI

## Abstract

CD36 is a type 2 cell surface scavenger receptor widely expressed in many immune and non-immune cells. It functions as both a signaling receptor responding to DAMPs and PAMPs, as well as a long chain free fatty acid transporter. Recent studies have indicated that CD36 can integrate cell signaling and metabolic pathways through its dual functions and thereby influence immune cell differentiation and activation, and ultimately help determine cell fate. Its expression along with its dual functions in both innate and adaptive immune cells contribute to pathogenesis of common diseases, including atherosclerosis and tumor progression, which makes CD36 and its downstream effectors potential therapeutic targets. This review comprehensively examines the dual functions of CD36 in a variety of immune cells, especially macrophages and T cells. We also briefly discuss CD36 function in non-immune cells, such as adipocytes and platelets, which impact the immune system via intercellular communication. Finally, outstanding questions in this field are provided for potential directions of future studies.

## Teaser

CD36 functions as both a signaling receptor and fatty acid transporter in various immune and non-immune cells. This review summarizes how its dual functions determine immune cell functions and fates, which contribute to common diseases including atherosclerosis and tumor progression.

## Introduction

The word “immunometabolism” appeared in the literature about a decade ago, prompted by the observations that metabolic diseases are often associated with dysregulated immune system (Mathis and Shoelson, 2011). It is an emerging research field that integrates cell metabolism and immunology. Thanks to many groundbreaking discoveries in the past decade, it is now widely recognized that cellular metabolism does more than provide energy and building blocks for de novo synthesis of macromolecules such as DNA, RNA, proteins, and lipids during immune cell activation. Many metabolic intermediates, in fact, are themselves critical signaling molecules leading to either pro- or anti-inflammatory responses (Galván-Peña et al., 2019; Mills et al., 2018; Tannahill et al., 2013). Similar to the dynamic nature of immune cell activation, cellular metabolic pathways are highly flexible as immune cells face different extracellular environments. Importantly, metabolic reprogramming from predominantly aerobic to anaerobic metabolism or vice versa often precedes and even drives immune cell differentiation, activation, and death (Chen et al., 2019; Ma et al., 2021; Mills et al., 2016), providing rationale for manipulating specific components of cell metabolism as a therapeutic strategy against many common diseases including cancer and atherosclerosis (O’Neill et al., 2016; Voss et al., 2021). A critical question in the field of immunometabolism is how cell metabolic pathways and immune cell differentiation/activation are integrated and how this integration is dysregulated under pathological conditions. One molecule that may hold a key to this question is CD36.

**Figure 1.**
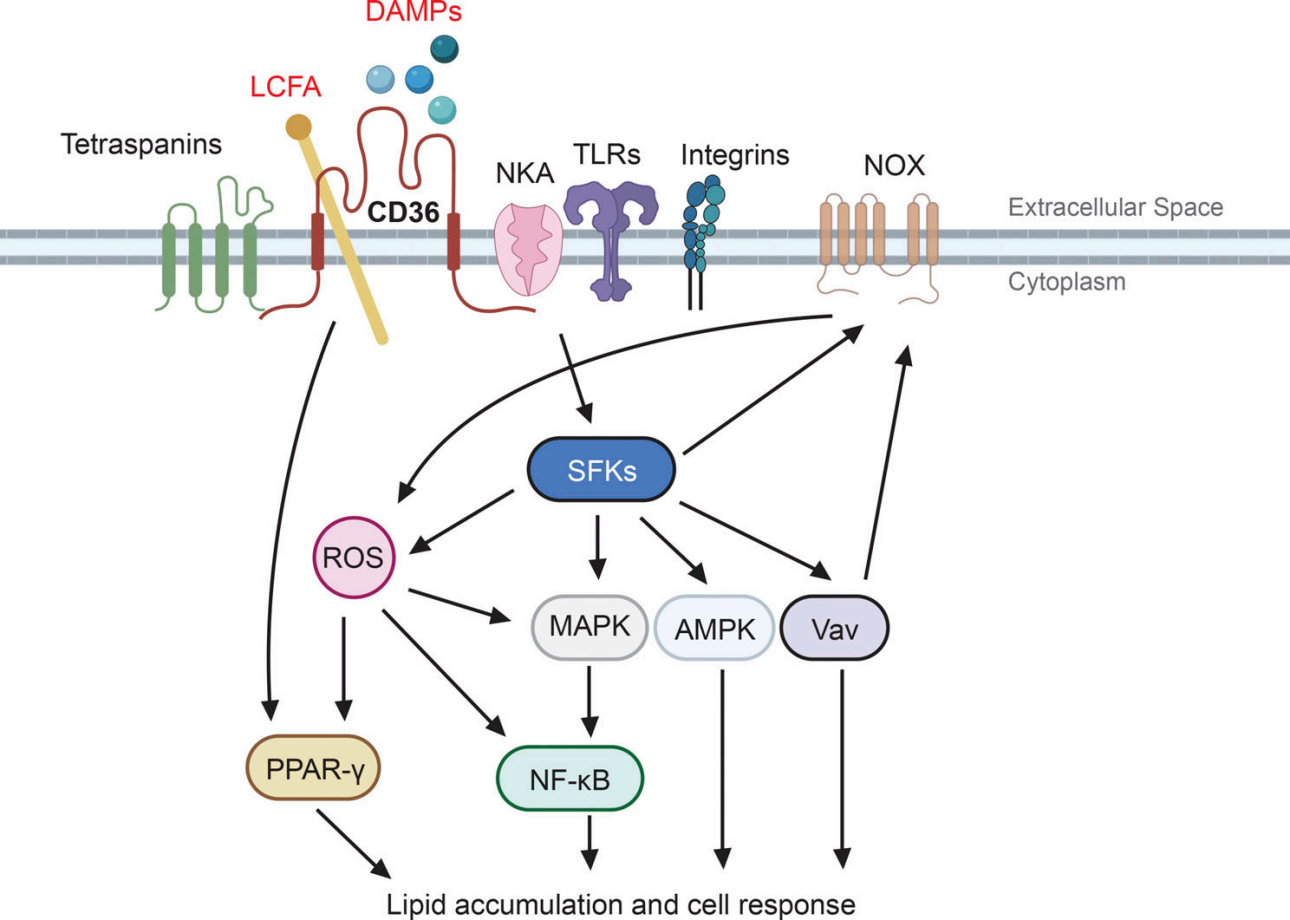
**CD36 functions as both a signal transducer and fatty acid transporter.** In response to extracellular signals such as DAMPs, CD36 assembles and interacts with other membrane receptors, including NKA, TLRs, integrins, and tetraspanins, forming distinct signaling complexes. The signaling complexes subsequently relay the signals to various downstream effectors including SFKs, MAPK, AMPK, guanine nucleotide exchange factor Vav, and the NOX family of nicotinamide adenine dinucleotide phosphate oxidases. Activation of these effectors leads to ROS production as well as transcription factor activation, including PPAR-γ and NF-κB. Meanwhile, CD36 also binds with LCFAs and facilitates their transport across the plasma membrane, which impacts fatty acid metabolism and may contribute to activation of the PPAR-γ pathway. The dual functions of CD36 eventually integrate, leading to different cellular responses such as migration, immune activation, differentiation, and cell growth/death.

CD36 is a heavily glycosylated 88-kD class B scavenger receptor expressed on the surface of a wide variety of innate and adaptive immune cells, including macrophages, monocytes, dendritic cells (DCs), and subsets of T and B cells, as well as many non-immune cells including platelets, immature erythrocytes, adipocytes, myocytes, certain specialized epithelial cells, and microvascular endothelial cells. CD36 binds to a large and diverse group of extracellular ligands falling into three broad categories—proteins containing thrombospondin structural homology repeat (TSR) domains, long-chain fatty acids (LCFAs), and molecules exhibiting molecular structures consistent with danger-associated or pathogen-associated molecular patterns (DAMPs and PAMPs; Fig. 1). Binding of CD36 to TSR domain proteins, including thrombospondin-1 and -2, mediates anti-angiogenic signaling in vascular endothelial cells and pro-thrombotic signaling in platelets (Chu et al., 2013; Roberts et al., 2010). This function is less well studied in immune cells, although macrophage CD36 may participate in thrombospondin-mediated regulation of latent TGFβ activation (Yehualaeshet et al., 1999).

This review will focus on CD36 function as a receptor for LCFAs and DAMPs/PAMPs as these are most relevant to innate and adaptive immunity (Silverstein and Febbraio, 2009; Wang et al., 2020). CD36 binding to LCFA was discovered by Harmon and Abumrad (1993) through expression cloning experiments, and their group and others subsequently showed that this function was critically important to facilitate fatty acid transport into fat and muscle cells (Coburn et al., 2000; Ibrahimi et al., 1999). CD36 orthologs in insects mediate sexual behavioral responses to fatty acid–based pheromones, and CD36 signaling in taste bud and enteric cells in response to fatty acids in food regulates food preference and neuro-enteric signaling in vertebrates (El-Yassimi et al., 2008; Laugerette et al., 2005). These studies highlight the importance of CD36 as both an LCFA transporter and a cellular signaling receptor.

Although the structure of CD36 has not been solved at the atomic level, structures of homologous proteins have been solved, and recent in silica and computational modeling studies have linked LCFA binding and transport to a putative binding pocket in the CD36 extracellular domain (Hsieh et al., 2016). The recognition site on CD36 for TSR domain proteins has been localized to a short 20-amino acid sequence in the CD36 extracellular domain (Frieda et al., 1995), and nuclear magnetic resonance studies identified specific amino acid residues in CD36 and the TSR domain that mediate binding (Klenotic et al., 2013). Peptides mimicking these domains block TSR protein binding to CD36 and impact tumor angiogenesis in mouse models (Hale et al., 2012). Binding sites in CD36 for DAMPs and PAMPs have not been identified with certainty, probably because these interactions are promiscuous and of low to moderate affinity.

Expression of CD36 is regulated at both the transcriptional and posttranslational levels, but regulation differs among different cell types. Similar to many other genes involved in lipid metabolism, CD36 expression in fat and muscle is upregulated by the nuclear hormone transcription factor PPAR-ɣ (Chui et al., 2005; Lim et al., 2006). In monocytes, CD36 is upregulated by PPAR-ɣ, as well as by cytokines including macrophage CSF, IL4, and IL10 that are involved in differentiation toward DCs and reparative “M2”-like phenotypes (Huh et al., 1996; Szatmari et al., 2004; Tontonoz et al., 1998). In contrast, LPS and dexamethasone downregulate expression (Yesner et al., 1996). In microvascular endothelial cells, CD36 is downregulated by lysophosphatidic acid (Ren et al., 2016). Glycosylation also impacts CD36 expression levels, as does lipid acylation of both the N-terminal and C-terminal intracellular domains (Hoosdally et al., 2009; Tao et al., 1996).

The CD36 gene in humans is highly polymorphic, and polymorphisms in the locus have been associated with levels of CD36 expression and with cardiovascular, thrombotic, and metabolic phenotypes (Ghosh et al., 2011; Melis et al., 2017; Momeni-Moghaddam et al., 2019). Surprisingly, CD36 null alleles are common in humans, with estimates in African and Asian populations as high as 26% (Fry et al., 2009; Hirano et al., 2003). The CD36 null state in humans appears at expected percentages based on the frequency of the null allele and is not known to be associated with decreased life expectancy or with risk of specific diseases, although limited studies have shown dysfunction in LCFA uptake in heart and muscle and perhaps increased risk of hypertension and metabolic syndromes (Miyaoka et al., 2001; Tanaka et al., 2001; Yanai et al., 2000). Mice deficient in CD36 are well studied and have normal life spans but significant increases in fasting levels of cholesterol, non-esterified free fatty acids, and triacylglycerol, along with decreased LCFA uptake into fat and cardiac and striated muscle (Febbraio et al., 1999). Under environmental or genetic conditions that promote chronic inflammation, oxidant stress, hyperlipidemia, and/or diabetes, CD36 deficiency has profound protective effect on atherosclerosis and thrombosis, pointing to important roles in homeostasis (Febbraio et al., 2000; Podrez et al., 2007).

Studies from our lab and others have indicated that CD36 links extracellular signals to intracellular fatty acid metabolism and redox metabolism in both innate and adaptive immune cells, impacting their fate and activation (Chen et al., 2019; Ma et al., 2021; Wang et al., 2020). Similar mechanisms operate in hematopoietic stem cells and tumor initiating cancer stem cells where CD36 expression promotes and maintains “stemness” by sensing extracellular ligands, including oxidized phospholipids (Hale et al., 2014), and by facilitating fatty acid uptake from surrounding adipose tissues to fuel fatty acid oxidation (Ye et al., 2016). In this review, we focus on how the dual functions of CD36 as a fatty acid transporter and cell signaling receptor control immune cell fate and how this impacts disease progression in cancer and atherosclerosis. Although we will mainly review its role in immune cells, we will briefly discuss how CD36 in non-immune cells may regulate immune cells via transcellular communication.

## The dual functions of CD36 as both a signal transducer and fatty acid transporter

Innate immune cell activation can be stimulated by a variety of molecules that express DAMPs or PAMPs (Chen and Nunez, 2010). Some, such as ATP, mitochondrial DNA, and DNA–histone complexes can be secreted or released by damaged or dying cells, some such as specific polysaccharides, glycolipids, and glycans are expressed on the surface of invading pathogens, and others can be generated by extracellular modifications of circulating or matrix proteins and lipids by oxidant stress, nitrative stress, hyperglycemia, and/or hyperlipidemia (Roh and Sohn, 2018). DAMPs and PAMPs represent extracellular alarm signals alerting nearby immune cells about surrounding tissue damage or pathogen invasion. Accordingly, immune cells express many cell surface pattern recognition receptors that bind to and sense specific portfolios of ligands; among these are the family of TLRs and several genetically unrelated families of scavenger receptors.

CD36 recognizes and binds with high affinity to specific oxidized phospholipid moieties found in oxidized low-density lipoproteins (oxLDL) and on the surface of some apoptotic cells (Greenberg et al., 2006; Nicholson et al., 1995; Podrez et al., 2000). It also binds to glycated proteins (so-called advanced glycated endproducts) formed in the presence of sustained hyperglycemia, such as seen in the setting of poorly controlled diabetes (Zhu et al., 2012) to pro-inflammatory S100 proteins released by activated neutrophils (Kerkhoff et al., 2001; Tondera et al., 2017), and amyloid β proteins found in Alzheimer brains (El Khoury et al., 2003). In addition to its interactions with DAMPs, CD36 also binds lipoteichoic acid on staph bacteria (Hoebe et al., 2005), glycans on certain fungi (Means et al., 2009), and proteins expressed on the surface of falciparum malaria–infected erythrocytes (Smith et al., 2003). DAMP/PAMP interactions with CD36 trigger an intracellular signaling cascade that begins with src-family kinase (SFK) activation and then involves specific MAPKs, Vav-family guanine nucleotide exchange factors, and transcriptional regulators (Chen et al., 2019; Li et al., 2010; Rahaman et al., 2006). Ultimately cytoskeletal reorganization, ROS production (Chen et al., 2019; Park et al., 2009), and the secretion of inflammatory cytokines ensues resulting in internalization of bound ligands, altered migratory behavior, and amplification of pro-inflammatory signals (Chen et al., 2019; El Khoury et al., 2003; Fig. 1).

CD36 has two transmembrane domains, and both intracellular domains are quite short (7 amino acids at the N-terminus and 13 amino acids at the C-terminus); neither of them have intrinsic kinase or phosphatase activity, scaffolding domains, or any binding sites for GTPases to relay signals (Silverstein and Febbraio, 2009). Therefore, to transduce signals to different downstream intracellular effectors CD36 must initiate assembly of a signalosome complex that includes intracellular and membrane protein partners, such as TLRs 2, 4, and 6 (Stewart et al., 2010; Triantafilou et al., 2006), Na/K-ATPase (NKA; Chen et al., 2015; Kennedy et al., 2013), β1/β2 integrins (Heit et al., 2013), and tetraspanins (Huang et al., 2011; Fig. 1). By assembling membrane-associated signaling complexes, CD36 also facilitates signaling by other receptor–ligand interactions. For example, cardiotonic steroids bind to the NKA α1 subunit, inducing its interaction with CD36/TLR4 and activation of NF-κB pathway, leading to pro-inflammatory responses in macrophages (Chen et al., 2017). Similarly, TLR1/2 signaling in the brain requires CD36 (Abe et al., 2010), and TLR4 signaling in macrophages is modulated by CD36. It is not clear how CD36 assembles different signaling partners when cells are facing different stimuli. It is possible that different ligands create a variety of CD36 conformational changes, or more likely that different ligands recruit different membrane partners into the signaling complex. Another likely possibility is that since CD36 localizes preferentially in cholesterol-rich membrane lipid rafts and perhaps in tetraspanin-regulated membrane microdomains, ligand binding could affect localization of CD36 and other signaling molecules so that they have higher chances of interacting with each other.

A unique feature that distinguishes CD36 from other pattern recognition receptors is its ability to facilitate LCFA transport. The LCFA trafficking function of CD36 was first identified by Harmon and Abumrad, who identified a 88-kD fatty acid binding protein on rat adipocytes as CD36 (Harmon and Abumrad, 1993). Subsequent studies showed that CD36 was induced during preadipocyte differentiation and facilitated LCFA uptake by cells (Abumrad et al., 1993). Later, its LCFA transport role was demonstrated in muscle cells (Ibrahimi et al., 1999; Van Nieuwenhoven et al., 1995), endothelial cells (Son et al., 2018), and immune cells, including macrophages (Chen et al., 2019) and T cells (Wang et al., 2020). Interestingly, binding of LCFA to CD36 is accompanied by intracellular signaling events and adjustment of lipid metabolism, including dissociation of the SFK Fyn from the CD36 signaling complex and enrichment of cytosolic liver kinase B1, which activates AMP-activated protein kinase (AMPK) pathway and upregulates fatty acid oxidation (FAO; Samovski et al., 2015). LCFA/CD36 binding modulates oxLDL binding/uptake and cellular cholesterol level (Jay et al., 2015; Vazquez et al., 2020). Thus, at least for the oxLDL/CD36 signaling pathway, evidence strongly indicates that the dual functions of CD36 as a signaling transducer and LCFA transporter are closely connected and integrated in a cell-type dependent manner (Fig. 1).

Multiple layers of cell-specific regulation of CD36 expression and function impact immune cells. At the transcriptional level, PPAR-γ:RXRα heterodimers bind to the promoter of the CD36 gene and positively control its transcription. The PPAR-γ pathway also upregulates genes involved in intracellular LCFA transport, including FABP4, and thereby plays an important role in homeostatic regulation of lipid uptake and metabolism in myocytes and adipose tissue. PPAR-γ in macrophages, however, is activated by oxLDL internalization, presumably due to intracellular delivery of bioactive lipids that can be metabolized to high affinity ligands for PPAR-γ. This then stimulates CD36 transcription creating a “feed forward” positive feedback loop that in the atherogenic arterial wall ultimately results in unchecked uptake of oxLDL and formation of foam cells and plaque (Nagy et al., 1998; Tontonoz et al., 1998). Besides endogenous ligands such as oxLDL, exogenous PPAR-γ ligands, including the thioglitazone class of drugs, also upregulate CD36 (Ballesteros et al., 2014; Kolak et al., 2007), perhaps contributing to the increase in cardiovascular risk associated with their use in patients with type 2 diabetes. Other transcription factors reported to regulate CD36 transcription in different cell types include C/EBPα in 3T3-L1 adipocytes (Qiao et al., 2008), activating transcription factor 2 in Ly6C^high^ monocytes (Raghavan et al., 2018), and signal transducer and activator of transcription (STAT)-5 in hepatocytes (Hosui et al., 2017), STAT1 in THP-1 human monocytic cell line (Kotla et al., 2017), and STAT3 in lymphocytic leukemia cells (Rozovski et al., 2018). Induction of CD36 expression by various transcription factors is generally accompanied by increase in lipid or lipoprotein uptake. At the posttranscriptional level, CD36 mRNA is a target of several microRNA (miRNA) such as miR-758-5p (Li et al., 2017), miR-34a (Lavery et al., 2016), miR-135a (Du and Lu, 2018), miR-181a (Du et al., 2018), and miR-182-5p (Qin et al., 2018). Interestingly, all these miRNA species target other metabolic enzymes or key regulators.

At the posttranslational level, CD36 is subjected to palmitoylation (Tao et al., 1996), ubiquitination (Smith et al., 2008), phosphorylation (Asch et al., 1993), glycosylation (Hoosdally et al., 2009), and lipid acylation (Kuda et al., 2013). These modifications are most often linked to cellular metabolic status, especially for palmitoylation, glycosylation, and acylation, since the metabolic intermediates are either directly or indirectly used as substrates for the posttranslational modifications. Thus, existing evidence strongly suggests that CD36 expression and function are coupled to cellular metabolism. In the following sections, we review current knowledge of how the dual functions of CD36 in immune cells determine cell fate.

## CD36 in innate immunity

### CD36 in macrophage activation, differentiation, and metabolic switch

**Figure 2.**
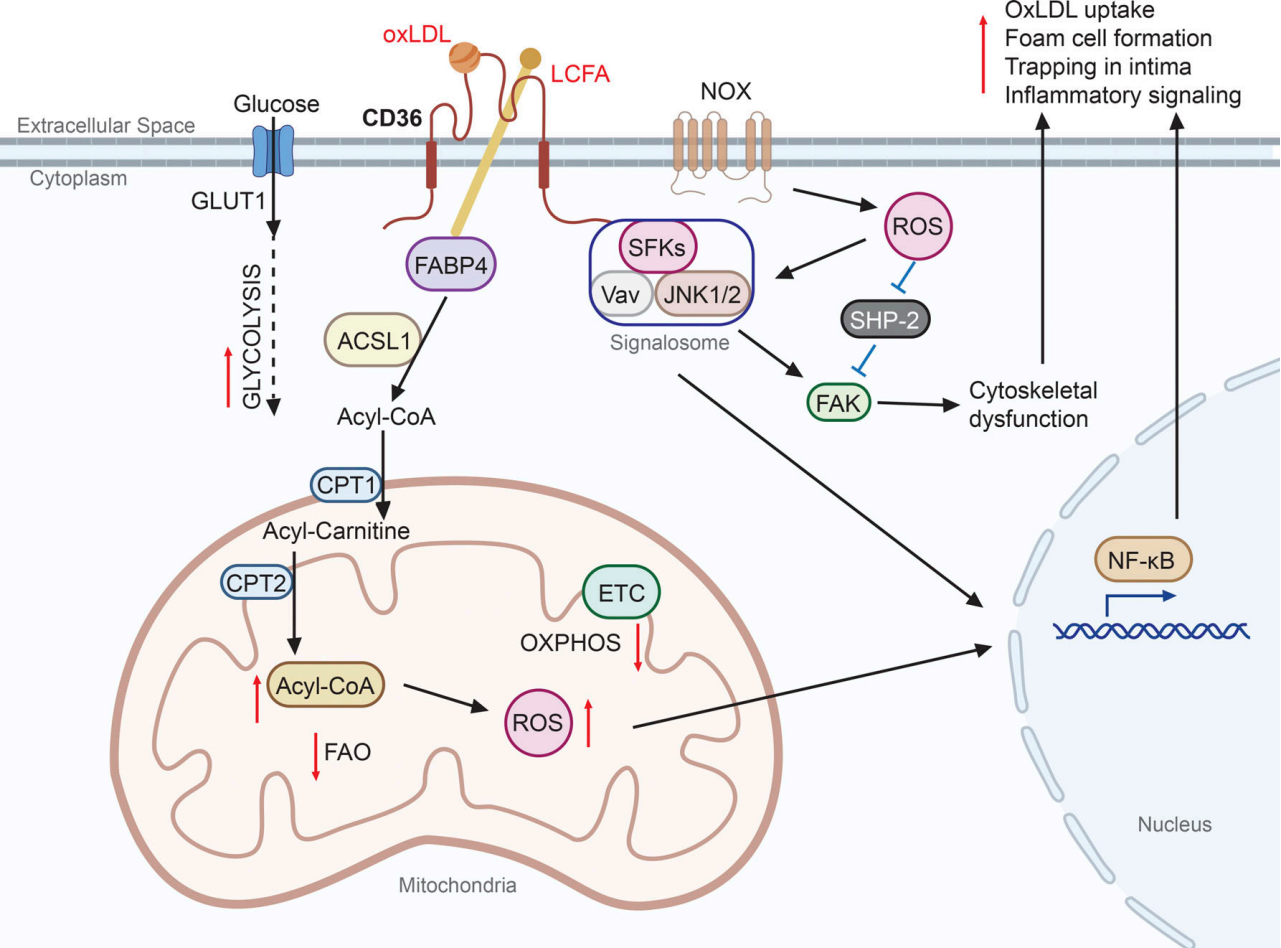
**CD36 mediates macrophage activation.** oxLDL binds to extracellular domains of CD36, which promotes both signaling cascades and lipid uptake. The CD36 cytosolic tail recruits a signalosome complex including SFKs, JNK1/2, and Vav, which is also enhanced by NOX-derived ROS. Both signalosome and ROS (by inhibition of SHP-2) activate focal adhesion kinase (FAK), which leads to dysregulated cytoskeletal dynamics. Meanwhile, extracellular LCFA is transported into the cell via CD36 and then into mitochondria matrix through a series of transport machinery interactions including FABP4, ACSL1, CPT1, and CPT2. LCFA influx to mitochondria facilitates a metabolic switch from OXPHOS to glycolysis, accompanied by a reduction in FAO and increase in ROS production. The signaling cascades and metabolic switch in combination results in NF-κB pathway activation and proatherogenic responses including pro-inflammatory activation, oxLDL uptake, foam cell formation, and trapping of macrophages in the neointima.

Although widely considered to be terminally differentiated cells, macrophages are actually highly flexible and display numerous distinct activation status in response to different stimulants (Mosser and Edwards, 2008). Alternative macrophage activation by IL-4 upregulates CD36 expression (Feng et al., 2000), FAO, and oxidative phosphorylation (OXPHOS) to fuel cell activation responses (Vats et al., 2006), and CD36-deficient macrophages display defective alternative activation (Huang et al., 2014). The microenvironment in which macrophages reside thus has a strong influence on their differentiation and activation status. For example, in the wall of an atherogenic artery where LDL particles translocate from the circulation through the endothelium and become trapped in the intima, the LDL becomes oxidized to form oxLDL. oxLDL binds to CD36 with high affinity, activating a signaling cascade, including SFKs, Vav, and JNK1/2, necessary for lipoprotein uptake and foam cell formation (Chen et al., 2015; Huang et al., 2011; Rahaman et al., 2006). CD36-mediated oxLDL uptake may also result in formation of intracellular cholesterol crystals or fibrils that stimulate macrophage activation through the NOD-, LRR-, and pyrin domain-containing protein inflammasome pathway, leading to secretion of the pro-inflammatory cytokine IL-1β (Sheedy et al., 2013). In microglial cells, CD36 can participate in activation of the inflammasome pathway in response to amyloid peptides, promoting inflammation in the brain, and perhaps contributing to the pathogenesis of Alzheimer’s disease (El Khoury et al., 2003; Sheedy et al., 2013). In addition, oxLDL/CD36 signaling leads to nicotinamide adenine dinucleotide phosphate oxidase (NOX)–derived ROS production, which subsequently inactivates Src homology-2–containing phosphotyrosine phosphatase (SHP-2). Combined with SFK activation, the net result is sustained activation of focal adhesion kinase and dysregulated cytoskeletal dynamics, which suppresses macrophage motility, contributing to trapping in the intima (Park et al., 2009). Moreover, expression of distinct LCFA transport machinery involving CD36, fatty-acid-binding protein 4 (FABP4), acyl-CoA synthetase 1 (ACSL1), and carnitine palmitoyltransferase complex 1 and 2 (CPT1 and CPT2) is induced by oxLDL, and these proteins work in sequence to transport exogenous LCFA into the mitochondria matrix. However, unlike the alternative activation pathway that stimulates FAO and OXPHOS, the oxLDL/CD36 axis suppresses both pathways and induces a metabolic switch to glycolysis for energy. Consequently, LCFA accumulate in mitochondria and facilitate ROS production, probably due to defects in inner membrane formation leading to electron leakage. Mitochondrial ROS subsequently activate the redox-sensitive NF-κB pathway and promote macrophage pro-inflammatory activation (Chen et al., 2019; Fig. 2). These examples demonstrate that CD36 upregulation is not always associated with FAO induction and that CD36 is able to support both anti- and pro-inflammatory activation. Determining how CD36-mediated lipoprotein/LCFA uptake is coordinated with intracellular LCFA trafficking and catabolism via FAO may shed light on mechanisms by which macrophages can be differentially activated by distinct extracellular signals.

As a receptor for PAMPs expressed on certain bacteria, yeast, and parasites, macrophage CD36 also participates in the innate immune response to infections. A forward genetic study in mice showed that absence of CD36 in the BalbC strain created a recessive phenotype of hypersensitivity to staphylococcus aureus infection (Hoebe et al., 2005). This phenotype was tracked to defective TLR2 signaling in response to specific diacylglceride species in the staph cell wall lipoteichoic acid. The ability of CD36 to partner with various TLRs including TLR2, TLR4, and TLR6 has since been shown to be an important and common theme in innate immunity. Interestingly, the staph hypersensitivity phenotype has not been seen in other mouse strains with CD36 deficiency, such as C57Bl6, nor has it been reported in humans with homozygous CD36 deficiency, suggesting that other genetic modifiers may be involved. The protozoan parasite *Leishmania amazonensis* is an important human pathogen that survives within IFN-ɣ–stimulated, inflammatory macrophages by localizing within large fusogenic parasitophorous vacuoles. Genetic studies in fly and mouse models identified CD36 as an important contributor to this pathway. CD36 localizes at sites where parasites contact the vacuole membrane, and CD36-deficient macrophages showed defective vacuole formation, suggesting that CD36 might be involved in vacuole biogenesis and fusion, perhaps through its role in lipid trafficking since cholesterol retention has also been shown to be necessary for vacuole formation/fusion (Okuda et al., 2016; Pessoa et al., 2019).

Macrophages within the microenvironment of malignant tumors (TME), so-called tumor-associated macrophages (TAMs), commonly display dysregulated lipid metabolism (Corn et al., 2020). Consistent with the mechanism described in previous paragraphs, a recent study reported that TAMs upregulated CD36 to increase LCFA uptake, which was accompanied by intracellular lipid accumulation and higher FAO. Additionally, elevated FAO promoted mitochondrial OXPHOS and ROS production, leading to STAT6 activation and downstream gene transcription that further promoted pro-tumor function (Su et al., 2020).

The TME also influences function of specialized immature myeloid cells known as myeloid-derived suppressor cells (MDSCs) through CD36 (Xin et al., 2021). MDSCs are a heterogenous population of myeloid cells characterized by their ability to suppress both innate and adaptive immune responses. Immune checkpoint blockade is an important clinical strategy for cancer therapy, but its efficacy is limited by resistance mechanisms, including the presence of an immunosuppressive TME (Wei et al., 2018). MDSCs constitute the largest population of suppressor cells in the TME and are thus an important barrier for effective cancer immunotherapy. Using single-cell RNA sequencing in a bilateral tumor model, Xin et al. (2021) showed that MDSCs from immune checkpoint blockade-resistant tumors displayed a higher fatty acid transport, FAO, and OXPHOS gene expression signature compared with cells from susceptible tumors. Transcription of these genes, along with CD36, is controlled by PPAR-γ, and the PPAR-γ–CD36 axis is downstream of a constitutively active oncogenic kinase, Pim1 (Meeker et al., 1987). Thus, the TME appears to take advantage of CD36 fatty acid transport functions to manipulate surrounding innate immune cells such as TAMs and MDSCs to avoid immune activation against cancer cells. Recent studies demonstrate that TME also affects adaptive immune cells including regulatory T (T_reg_) and CD8 T cells, which facilitates tumor growth. The next section will elaborate how TME regulates T cells through CD36.

Adipose tissue in the setting of obesity is another microenvironment where metabolic regulation by CD36 plays an important role. Adipocytes constitute the majority of the adipose tissue cells, but a variety of immune cells including macrophages, DCs, eosinophils, innate lymphoid cells, T cells, and natural killer (NK) cells are also present (Kane and Lynch, 2019). Accumulating evidence shows that adipocytes, by secreting adipokines or extracellular vesicles, constantly communicate with their surrounding immune cells, especially adipose tissue macrophages (ATMs), and regulate immune function (Flaherty et al., 2019; Kennedy et al., 2011; Kosteli et al., 2010; Suganami et al., 2005). In fact, adipose tissue is implicated in dysregulation of the immune system seen in common diseases such as obesity, type II diabetes, atherosclerosis, and cancers (Boutens and Stienstra, 2016; Fantuzzi and Mazzone, 2007; Lengyel et al., 2018).

CD36 is highly expressed in both adipocytes and ATMs, distinct from other scavenger receptors such as SR-A and LOX-1, which show <10% of the mRNA levels compared with CD36 (Rasouli et al., 2009). This large difference in adipose tissue expression is not surprising as CD36 plays a critical role in LCFA uptake by adipocytes (Coburn et al., 2000). Yet, this unique expression pattern of CD36 within the adipose tissue provides a feed-forward inflammatory paracrine loop between adipocytes and ATMs in obese adipose tissue and hyperlipidemia conditions (Kennedy et al., 2011). Diet-induced obesity in mice significantly enhanced inflammatory activation of ATMs and promoted oxidative stress, and adipose tissue from *cd36* null mice showed decreased activation of ATMs and adipocytes and less insulin resistance (Kennedy et al., 2011), suggesting that a paracrine loop between adipocytes and ATMs facilitated chronic inflammation dependent on surface CD36 expression in both cell types (Kennedy et al., 2011). At the subcellular organelle levels, it was shown that adipocyte CD36-mediated signaling resulted in excess calcium release from the ER to lysosomes, which impaired lysosome functions and promoted inflammation (Luo et al., 2020). Thus, CD36 not only influences intracellular lipid metabolism and pro-inflammatory signaling, but also participates in intercellular communication. Interestingly, adipocytes can communicate with immune cells beyond adipose tissues via extracellular vesicles, and CD36 is also involved in this pathway (Yan et al., 2021). We will elaborate the story of extracellular CD36 in section 6.

### CD36 in circulating monocytes

Compared to macrophages, the role of CD36 in circulating monocytes is less well understood (Moore and Tabas, 2011). Monocyte CD36 may be beneficial for vascular homeostasis, and so-called “nonclassical” Ly6C^−^Cx_3_CR1^high^ monocytes circulate for prolonged time periods patrolling along the vascular endothelium to promote vascular maintenance. Marcovecchio et al. (2017) recently showed that CD36 is essential for the function of nonclassical monocytes by regulating patrolling activity during early stage of atherosclerosis. The adaptor protein DAP12 and SFKs facilitate the patrolling function, and deleting CD36 in these cells decreased oxLDL uptake and significantly reduced patrolling speed and distance. Interestingly, deleting a different scavenger receptor, SR-A, did not reduce oxLDL uptake or numbers of patrolling cells, but SR-A deficient cells had defects in patrol speed and distance (Marcovecchio et al., 2017). CD36-mediated oxLDL uptake and downstream signaling for patrolling activity therefore could not be fully compensated by other scavenger receptors. Whether CD36 regulation of metabolic status is involved in the patrolling function of non-classical monocytes remains to be determined.

## CD36 regulation of adaptive immunity and B and T cell metabolism

### CD36 in T cell activation and survival/death

**Figure 3.**
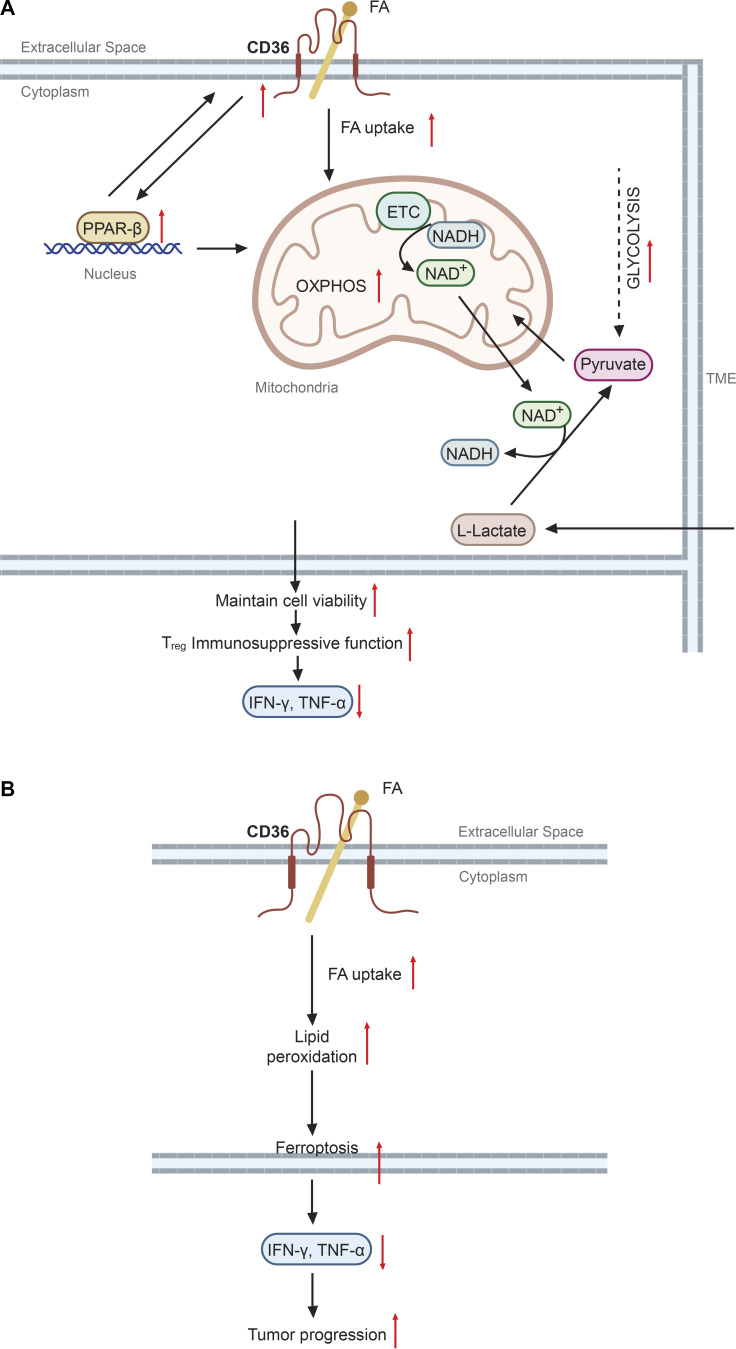
**CD36 mediates differential responses in T cell subsets. (A)** In T_reg_ cells, CD36 is upregulated in the TME and facilitates fatty acid (FA) uptake, which subsequently stimulates PPAR-β signaling. Both CD36-mediated fatty acid uptake and PPAR-β signaling support mitochondrial OXPHOS. As a consequence, NAD^+^ is continuously produced from nicotinamide adenine dinucleotide (NADH) through the mitochondrial electron transport chain (ETC). T_reg_ cells utilize this NAD^+^ pool for metabolism of L-lactate to pyruvate so that they are better adapted to a high-lactate TME and maintain viability and immunosuppressive functions. **(B)** In TME CD8^+^ T cells, CD36-mediated fatty acid uptake leads to lipid peroxidation and ferroptosis so that these cells are not able to produce cytotoxic cytokines.

The role of CD36 in T cell metabolism and function has drawn increasing attention in the context of cancer immunotherapy. Although the lipid-rich TME has been linked to immunosuppression (Beloribi-Djefaflia et al., 2016), the precise mechanisms underlying how accumulation of lipids regulates an anti-tumor immune response remain largely unknown. The discovery of CD36-expressing T_reg_ and CD8^+^ cytolytic T cells has shed some new light on this issue. Wang et al. (2020) found selective upregulation of CD36 in intra-tumoral T_reg_ cells in both human cancers and preclinical murine tumor models. Using an elegant T_reg_-specific CD36 deletion model, they demonstrated selective abrogation of abundance and suppressive activity of intra-tumoral T_reg_ cells. Targeting CD36 with a mAb also diminished T_reg_-mediated immunosuppression and CD36 blockade, either alone or in combination with anti–PD-1 treatment, and induced enhanced antitumor immunity (Wang et al., 2020; Fig. 3 A).

Two recent studies also found impaired function of CD36 in CD8^+^ T cells in the lipid-laden TME (Ma et al., 2021; Xu et al., 2021). Both studies showed convincing evidence of the intrinsic function of CD36 in taking up oxidized lipids and polyunsaturated fatty acids from the TME, which was intimately associated with impaired cytokine production and effector function. Consistently, CD36 deletion in CD8^+^ T cells reduced lipid uptake and increased antitumor immunity (Fig. 3 B). These studies collectively suggest that blocking CD36 on T_reg_ cells and/or CD8^+^ T cells could be a therapeutic approach to boost anti-tumor immunity.

Mechanistically, intra-tumoral T_reg_ cells upregulate CD36 resulting in increased fatty acid uptake, which stimulates PPAR-β signaling. Both CD36-mediated fatty acid uptake and PPAR-β signaling support mitochondrial health and continuous production of nicotinamide adenine dinucleotide (NAD^+^) though electron transport chains. This NAD^+^ pool is used to metabolize L-lactate to pyruvate so that T_reg_ cells are adapted to a high-lactate TME for better survival and immunosuppressive functions (Angelin et al., 2017; Wang et al., 2020; Fig. 3 A). In CD8^+^ T cells, CD36-mediated lipid uptake induces lipid peroxidation, which leads to the reduction of cytokine production and increased ferroptosis (Fig. 3 B). Given that glutathione peroxidase 4 (GPX4), an antioxidant enzyme, can rescue cells from ferroptosis by degrading lipid peroxides (Yang et al., 2014), Xu et al. (2021) further showed that GPX4 overexpression boosted CD8 effector T cell function and tumor control. Although it remains unclear how expression of CD36 on tumor-infiltrating T cells is regulated, this tissue selectivity provides a unique opportunity to develop promising strategies for metabolically targeting TME without significantly altering systemic tissue homeostasis.

### CD36 in B cell antigen response and cell death

Compared to macrophages and T cells, the functions of CD36 in B cells are less well studied. This may be due to the early reports that CD36 was only detected in a very small subset (∼3%) of B cells from normal human peripheral blood (Rutella et al., 1999). Later, more sophisticated studies in mice demonstrated that CD36 expression was mainly restricted to marginal zone B cells and was not detected or only barely detected in B1 and follicular B cells. Expression, however, was rapidly induced on follicular B cells by TLR and CD40 stimulation and this was accompanied by higher fatty acid uptake, lipid peroxidation, and ferroptosis when the antioxidant enzyme Gpx4 was not present (Muri et al., 2019). CD36^−/−^ mice appear normal in B cell development but showed reduced response to *Streptococcus pneumoniae*, producing less phosphocholine-specific IgM and IgG, indicating involvement of CD36 in B cell functions (Won et al., 2008). Another recent study demonstrated that CD36 mediated B cell autophagy and was colocalized with autophagosome membrane protein MAP1LC3/LC3. B cells deficient in CD36 significantly decreased plasma cell formation, activation, proliferation, and mitochondria mass as well as OXPHOS, further validating the essential role of CD36 in B cell metabolism and humoral responses (He et al., 2021). Together, these studies suggest that CD36 is critically involved in B cell fatty acid metabolism, bioenergetics, and cellular processes (e.g., autophagy), which in turn affect B cell activation and survival/death status.

### CD36 in DC function

CD36 is highly expressed on subsets of DCs, including so-called “immature” DCs and CD8^+^ DCs (Albert et al., 1998). This is not surprising given the roles of DCs in phagocytosis and efferocytosis of apoptotic tumor cells and cells infected with viruses or other intracellular pathogens, and studies showing that cytokines known to induce DC differentiation from hematopoietic precursors (e.g., IL-4 and macrophage CSF) are potent inducers of CD36 expression in monocyte/macrophages (Yesner et al., 1996). Studies suggest that CD36-mediated efferocytosis or phagocytosis may participate in DC presentation of tumor antigens, viral antigens, and malarial antigens through both MHC class I and class II systems, and therefore may play a role in modulating T cell and B cell responses (Urban et al., 2001). It is reasonable to hypothesize that the excess intracellular lipid load resulting from CD36-mediated uptake of infected and apoptotic cells could influence metabolic pathways. While little direct data are currently available on this topic, some recent studies are suggestive (Zhang et al., 2019).

Autophagy is known to regulate metabolic pathways and has also been implicated in viral antigen presentation on MHC II. Oh and Lee (2019) showed that deletion of a critical autophagy gene, *Atg-5*, in mouse DCs was associated with increased CD36 expression, increased capacity to internalize apoptotic tumor cells, increased intracellular lipid accumulation, and reduced CD4 T cell priming. Blockade of CD36 in this model ameliorated the increased phagocytosis and restored CD4 T cell priming, consistent with a link between CD36, lipid accumulation, and DC function (Oh and Lee, 2019). In a different study, Hu et al. (2021) used a mouse model of hypertriglyceridemia and showed that DCs isolated from these mice had increased intracellular free fatty acids, increased ROS, increased expression of PD-L1, TGFβ, and NKG2D ligands, and enhanced NK cell suppressor activity. When CD36 levels were decreased by genetic deletion of one allele, the NK suppressor function was lost (Hu et al., 2021). These studies suggest that CD36-mediated lipid accumulation could alter DC metabolism and promote T cell and NK cell immunosuppression.

## CD36 in non-immune vascular cells and the potential role in immune regulation

### CD36 in platelet crosstalk with immune cells

While blood platelets are critically important for maintaining normal hemostasis and participate in pathological thrombosis, there is growing evidence of their contribution to inflammation and immunity (Ali et al., 2015). Platelets express Fc receptors, complement receptors, and CD40 ligand, and the platelet secretome contains numerous immune-regulatory molecules including chemokines, cytokines, complement components, and growth factors. As noted previously, CD36 signaling contributes to platelet hyperactivity under conditions of hyperlipidemia and chronic inflammation which not only promotes thrombosis, but also facilitates platelet–monocyte interactions (Lee et al., 2019; Podrez et al., 2007; Yang et al., 2017). These interactions facilitate monocyte adhesion to vascular endothelium and trafficking into inflamed tissues. Monocyte/macrophage phagocytosis of adherent platelets with their abundant lipid-rich membranes may also contribute to foam cell formation and inflammation (Badrnya et al., 2014). Platelet ROS generation is essential in CD36-mediated platelet activation (Yang et al., 2017), but whether metabolic switch, metabolic changes, and/or mitochondrial dysfunction are involved remains to be determined.

### CD36 in vessel wall cell biology

CD36 is expressed in microvascular endothelial cells and negatively regulates angiogenesis in response to protein ligands containing TSR domains, including thrombospondin-1 and 2 and vasculostatin (Chu et al., 2013; Simantov and Silverstein, 2003). Whether the fatty acid transport functions of CD36 are linked to its anti-angiogenic signaling and whether they affect endothelial cell interaction with immune cells is largely unknown. Endothelial CD36 has, however, been shown to mediate fatty acid uptake into heart, skeletal muscle, and brown adipose tissue, which influences glucose utilization and insulin sensitivity (Son et al., 2018), raising the possibility that endothelial CD36 could regulate fatty acid uptake by innate and adaptive immune cells in lymphoid tissues and/or in inflammatory microenvironments.

CD36 is also expressed in vascular smooth muscle cells (VSMCs), although at a much lower level than striated and cardiac muscle. Similar to macrophages and platelets, VSMCs generate ROS and produce inflammatory cytokines through a CD36 signaling cascade when stimulated by oxLDL. This pathway promotes neointimal hyperplasia in response to vascular injury and involves the specific SFK Fyn, which mediates phosphorylation and degradation of Nrf2, a key transcription factor controlling expression of anti-oxidant genes (Li et al., 2010). CD36 may also complex with TLR4 and urokinase receptor in response to oxLDL, mediating proinflammatory cytokine production through NF-κB activation (Kiyan et al., 2014). Whether CD36 fatty acid transport and metabolic regulatory functions are involved in VSMC function awaits further investigation.

### CD36 in cancer biology

There is considerable interest in the potential roles of CD36 in tumor biology. Studies have suggested that CD36 may promote tumor growth, metastasis, chemotherapy resistance, and resistance to checkpoint inhibitor immunotherapy, and that CD36 could be a therapeutic target in cancer. Many tumor cells express CD36, including certain types of breast cancer, some melanomas, renal carcinomas, and gliomas, and in these settings CD36 may be enriched in tumor initiating cells, or so-called cancer stem cells. Its function in regulating metabolism may be essential in maintaining “stemness” and thus may contribute to chemo and radio resistance as well as metastatic potential (Feng et al., 2019; Hale et al., 2014; Martini et al., 2021). As implied by the previous sections in this review, the biology of CD36 within tumors is extraordinarily complex, and sorting out specific roles of the various cells within the TME that express CD36, including vascular endothelium, TAMs, MDSCs, T_reg_ cells, and CD8^+^ T cells requires sophisticated techniques in experimental animal model systems. Such data clearly show that CD36 function as a regulator of LCFA uptake and cellular metabolism in T cells can inhibit effector functions and contribute to escape from immune surveillance and resistance to checkpoint inhibitor immunotherapies. Similarly, CD36-mediated uptake of lipids and upregulation of fatty acid oxidation in TAMs and MDSCs may promote a protumor environment. These findings suggest that blocking CD36 could have anti-tumor activity, particularly in the setting of immunotherapy. On the other hand, endothelial cell CD36 may have anti-tumor activity by interfering with tumor angiogenesis. Most of this literature is based on mouse models and in vitro experiments, so much remains to be determined regarding the validity of CD36 as a target in human malignancies.

## Extracellular CD36: The potential role of soluble/circulating CD36 in immune regulation

Extracellular CD36, often referred to as “soluble CD36,” detected in human plasma by ELISA or Western blot analysis, has been shown in many studies to be elevated in patients with type II diabetes (fivefold compared with the lean control subjects and two- to threefold compared with the obese subjects) and to be inversely correlated with insulin-stimulated glucose clearance and positively correlated with fasting plasma insulin, glucose, and body mass index (Handberg et al., 2006). Circulating CD36 levels were positively correlated with severity of insulin resistance and atherosclerosis in several human population studies (Handberg et al., 2010; Handberg et al., 2008), and it was postulated that circulating CD36 could be used as a marker of metabolic syndromes (Koonen et al., 2011). Consistent with these population studies, a recent study of hyperlipidemic, atherogenic mice showed circulating CD36 levels fourfold higher than control animals. However, not all reported human studies have yielded the same results, possibly related to different methods for isolating and measuring the circulating CD36 (Alkhatatbeh et al., 2016).

The term soluble CD36 is misleading, as more recent studies showed that immune-detectable plasma CD36 is actually associated with circulating microvesicles (MVs; Alkhatatbeh et al., 2013). MVs are 200–1,000-nm-diameter membrane-bound particles that bud off the plasma membrane of apoptotic cells, activated platelets, activated leukocytes, and activated vascular cells. The cell type of origin of circulating CD36 in humans is poorly understood, although one study reported that CD36-containing MVs expressed markers of platelets, monocytes, erythrocytes, and endothelial cells, suggesting multiple cellular sources (Alkhatatbeh et al., 2013). Mechanistically, OxPC_CD36_, the CD36 ligand found in oxLDL, triggered release of CD36 from endothelial cells, macrophages, and adipocytes (Biswas et al., 2021). MVs carry intracellular cargo, including miRNAs, cytokines, and chemokines, that are unique to their cell of origin and can “deliver” this cargo to inflammatory and immune tissues when internalized by phagocytes.

Another recent study showed that a fraction of circulating CD36 may be in exosomes, smaller (50–200-nm diameter) membrane-bound particles derived from intracellular membrane compartments. Adipocytes treated with palmitic acid actively sort CD36 into exosomes for secretion to the circulation. This process is regulated by AMPKα, an important master regulator of cellular metabolism. Downregulation of AMPKα in adipocytes led to enhanced TSG101-mediated exosome biogenesis and CD36 sorting into the multivesicular bodies, the intracellular precursors of exosomes. Interestingly, CD36-containing exosomes were endocytosed by hepatocytes, which induced lipid accumulation and inflammation and facilitated nonalcoholic fatty liver disease (Yan et al., 2021). Thus, emerging evidence suggests that intercellular CD36 transfer through extracellular vesicles may be a novel mechanism for regulation of cellular lipid uptake. However, whether functional CD36 transfer exists in humans is not known, nor is it known if it contributes to metabolic switch and immune regulation.

## Conclusions, common themes, and perspectives

Decades of studies have shown that CD36 is a critical bridge linking inflammation to cellular metabolism. By serving as a DAMP/PAMP receptor in both immune and non-immune cells, CD36 mediates intracellular signaling events including kinase cascades, ROS production, and transcription factor activation to promote inflammation. By trafficking LCFA and being involved in efferocytosis, CD36 regulates multiple metabolic pathways. The dual functions appear to be unique to CD36 compared with other innate receptors such as TLRs, SR-A, LOX-1, and SR-BI. Recent findings have emphasized the critical role of CD36 dual functions in determination of immune cell fate in a cell type-dependent manner, e.g., M1/M2 macrophage differentiation (Huang et al., 2014), and T cell survival/ferroptosis (Ma et al., 2021; Wang et al., 2020; Xu et al., 2021). However, many findings are based on in vitro cultured cells and/or rodent models. We still know very little of how CD36 dual functions lead to different cell fate in distinct immune cell types and how these functions are dysregulated in humans with various diseases. For clarification of specific experimental settings from previous studies, we organized them in Table 1.

**Table 1.** Experimental settings on immune cell function/fate that are associated with CD36 dual functions

Cell type	Cell environment	Species	Cell function/Fate	Reference
Monocytes	In vitro culture	Human	Differentiation to macrophages	Huh et al. (1996)
Monocytes	In vitro culture	Human	Differentiation to DCs	Szatmari et al. (2004)
Macrophages	In vitro culture	Human and mouse	Phagocytosis	Greenberg et al. (2006)
Microglia and macrophages	In vitro culture	Mouse	Cytokine, chemokine secretion and ROS production	El Khoury et al. (2003)
Macrophages	In vitro culture	Human and mouse	Cell motility reduction	Park et al. (2009)
Macrophages	In vitro culture	Mouse	M2-like polarization	Vats et al. (2006)
Macrophages	In vitro culture	Mouse	Inflammasome activation and IL-1β secretion	Sheedy et al. (2013)
DCs	In vitro culture	Human	Efferocytosis	Urban et al. (2001)
Cancer stem cells	Tumor	Human and mouse	Self-renew	Hale et al. (2014)
Leukemic stem cells	Gonadal adipose tissue	Human and mouse	Evade chemotherapy	Ye et al. (2016)
Nonclassical monocytes	Peripheral blood vessels	Mouse	Patrolling along blood vessels	Marcovecchio et al. (2017)
Macrophages	Aorta	Mouse	Foam cell formation	Febbraio et al. (2000); Rahaman et al. (2006)
Macrophages	Aorta	Human and mouse	Mitochondrial ROS production, glycolytic switch	Chen et al. (2019)
Macrophages	Tumor	Human and mouse	Fatty acid oxidation	Su et al. (2020)
Macrophages	Adipose tissue	Mouse	Inflammatory paracrine loop	Kennedy et al. (2011)
MDSCs	Tumor	Human and mouse	Immunosuppression	Xin et al. (2021)
T_reg_ cells	Tumor	Human and mouse	TME adaptation and immunosuppression	Wang et al. (2020)
CD8 T cells	Tumor	Human and mouse	Ferroptosis	Ma et al. (2021); Xu et al. (2021)
B cells	Blood	Human and mouse	Autophagy and humoral immune responses	He et al. (2021)

At the cellular level, CD36 is regulated through both transcriptional and posttranslational mechanisms during immune cell differentiation and activation, and the tissue microenvironment of CD36-enriched cells impacts their metabolic status and function as revealed by single-cell RNA sequencing. For example, in adipose tissues, CD36 is highly expressed in a subset of lipid-associated macrophages that are essential for protecting adipose tissues from inflammation and metabolic dysregulation (Jaitin et al., 2019). Similarly, in injured muscles, CD36 is enriched in a subpopulation of repair-type macrophages for muscles regeneration (Patsalos et al., 2022). However, in aorta tissues, CD36-enriched Trem2^+^ foam cells contribute to atherosclerotic plaque development (Cochain et al. [2018] and our unpublished data). To add to the complexity, CD36 can also exist in the extracellular vesicles, and its amount is regulated by intracellular vesicle biogenesis, trafficking, and secretion from various cell types. Regardless of its regulation mechanism, CD36 expression appears to be highly correlated to immunometabolic status and contributes to common diseases such as atherosclerosis and tumor development. Moreover, the dual functions of CD36 in determination of cell fate are likely applied to other non-immune CD36-expressing cells such as cancer stem cells. Therefore, CD36 is a promising target for therapeutic intervention. However, its biology is extraordinarily complex, and many important questions remain, including: How are the signaling and lipoprotein/LCFA trafficking dual functions coordinated and integrated? How broadly do the dual functions contribute to metabolic regulation and cell differentiation, activation, and survival across the spectrum of CD36-expressing cells? Mechanistically, how do the dual functions lead to different cell fates in different cell types? How well will murine models predict human biology of CD36 dual functions?
